# Undifferentiated Pleomorphic Sarcoma of the Duodenal Papilla: A Rare Case and Worth Discussing History

**DOI:** 10.3389/fsurg.2022.926003

**Published:** 2022-07-06

**Authors:** Jianlong Wang, Bin Liu, Jiachao Hou, Tao Li

**Affiliations:** ^1^ Department of General Surgery, The Second Hospital of Hebei Medical University, Shijiazhuang, China; ^2^ Central Laboratory, The Second Hospital of Hebei Medical University, Shijiazhuang, China

**Keywords:** undifferentiated pleomorphic sarcoma, duodenal papilla, duodenoscopy, duodenal papillectomy, case report

## Abstract

**Background:**

Undifferentiated pleomorphic sarcoma (UPS) is a malignant tumor that originates in the mesenchymal tissue and is common in the extremities and retroperitoneum. Primary UPS of the duodenal papilla is rare and a distinct clinical entity.

**Case presentation:**

In this report, a 48-year-old Chinese man was admitted to our hospital with symptoms of melena. The patient underwent choledochectomy and choledochaljejunostomy for obstructive jaundice 8 years before admission. Endoscopic examination after admission confirmed a mass located at the duodenal papilla. Then, the duodenal papilla and tumor resection were performed, and the histopathology report confirmed the diagnosis of UPS. The patient refused further treatment and died 2 months later due to local recurrence and intrahepatic metastasis.

**Conclusions:**

It is rare that the mass in the duodenal papilla is diagnosed as UPS. The unpredicted behavior of these tumors warrants a careful plan considering their indolent nature and possible recurrence and metastasis. The prognosis was poor despite the early complete resection.

## Introduction

Undifferentiated pleomorphic sarcoma (UPS) is the most common soft tissue sarcomas in the elderly (1, 2). It is a neoplasm considered to originate from primitive mesenchymal cells, arising from soft tissue or bone, usually in the extremities or retroperitoneum (3). The Vater papilla region is uncommon and primary duodenal papilla UPS is exceedingly rare, with only one case confirmed in the literature to date (Table 1). At present, the epidemiology, diagnosis, and treatment of this disease remain unclear (4–6). The following case documents a primary lesion of the duodenum papilla, and its biological behavior characterizing a worth discussing history and an extremely poor prognosis.

**Table 1:** Up-to-date review of cases of UPS around duodenal ampulla.

First author	Year	Age	Sex	Symptoms	Location	Treatment	Follow-up (months)
Margueles et al. (7)	1976	22	F	Postprandial pain, nausea	Head of pancreas	Pancreaticoduodenectomy	17 (alive)
Pascal et al. (8)	1989	39	M	Abdominal mass, reflux esophagitis	Head of pancreas	Pancreaticoduodenectomy	0 (died)
Suster et al. (9)	1989	71	M	Epigastralgia, nausea, jaundice	Head of pancreas	Pancreaticoduodenectomy	No-follow
Haba et al. (10)	1996	70	M	Vomiting, abdominal pain	Head of pancreas	Pancreaticoduodenectomy	22 (alive)
Mai et al. (11)	2002	71	F	Jaundice	Head of pancreas	Pancreaticoduodenectomy	24 (died)
Darvishian et al. (12)	2002	74	M	Accidentally discovered	Head of pancreas	Pancreaticoduodenectomy	4 (alive)
Yu et al. (13)	2008	67	M	Weight loss	Head of pancreas	Pancreaticoduodenectomy	11 (died)
Jarry et al. (14)	2010	45	M	Jaundice	Head of pancreas	Multidisciplinary treatment	11 (alive)
Sanei et al. (15)	2016	72	F	Abdominal pain	Head of pancreas	Pancreaticoduodenectomy	22 (alive)
Gilman et al. (16)	1986	29	F	Peritonitis	Duodenum	Pancreatoduodenectomy	0 (died)
Asai et al. (17)	1987	61	M	Epigastralgia	Duodenum	Resection	21 (alive)
Farinon et al. (18)	1999	61	F	Gastrointestinal bleeding	Duodenum	Pancreatoduodenectomy	2 (died)
Wang et al. (19)	2005	61	M	Gastrointestinal bleeding, weight loss	Duodenum	Pancreatoduodenectomy	24 (alive)
Tanaka et al. (17)	2005	53	M	Epigastralgia, fever	Duodenum	Chemotherapy	5 (died)
Makni et al. (20)	2011	63	M	Gastrointestinal bleeding, weight loss, vomiting	Duodenum	Gastroenteroanastomosis	4 (died)
Yuhei et al. (17)	2016	69	M	Epigastralgia, pancreatitis	Duodenum	Pancreatoduodenectomy	10 (alive)
Giuliani et al. (21)	2013	67	M	Jaundice	Duodenal papilla	Radiotherapy	No-follow
Present case	2018	48	M	Gastrointestinal bleeding	Duodenal papilla	Resection of tumor and duodenal papilla	2 (died)

*UPS, undifferentiated pleomorphic sarcoma; M, male; F, female.*

## Case Presentation

**Figure 1:**
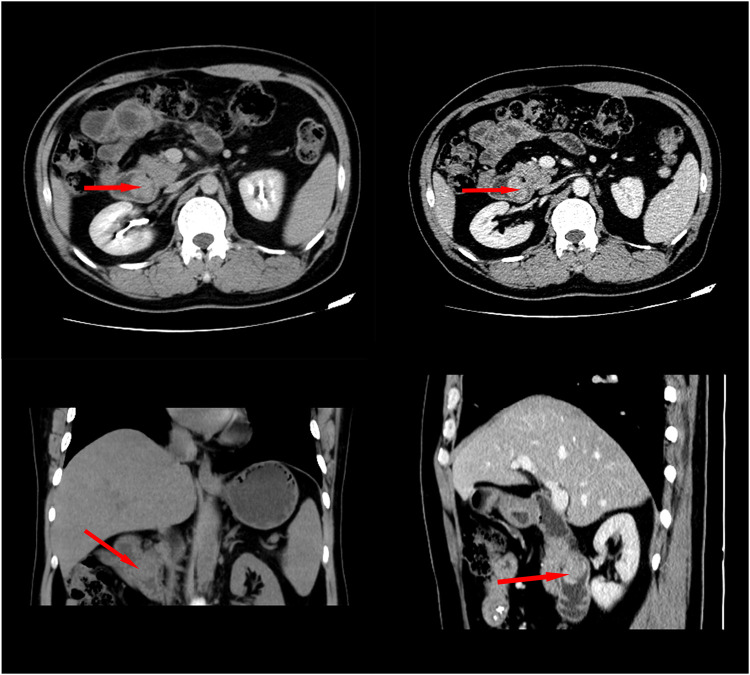
Contrast-enhanced CT showed that the tumor was located in the duodenal cavity (approximately 1.8 cm × 2.2 cm) without penetrating the duodenal wall.

**Figure 2:**
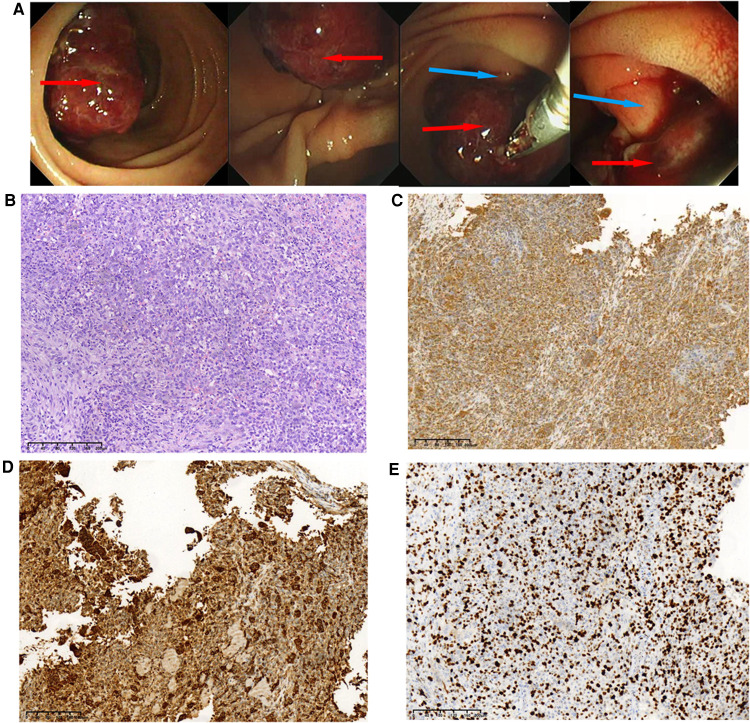
Duodenoscopic images of the tumor and pathological results of endoscopic biopsy during hospitalization. (**A**) Duodenoscopic images of the tumor; the red arrow indicates tumor tissue, and the blue arrow indicates duodenal papilla. (**B**)Histopathological examination of the biopsy (H&E staining) of the tumor, original magnification, ×100. These cells were positive for Vimentin, ×100 (**C**), CD68, ×100 (**D**), and Ki-67 was about 50% upon immunostaining, ×100 (**E**).

A 48-year-old Chinese man was admitted with melena (hemoglobin = 64 g/L). Contrast-enhanced CT scans showed that the mass was located in the descending duodenum, a cross section of approximately 1.8 cm × 2.0 cm. The values of plain and three-phase contrast-enhanced CT scans were about 35/90/90/73HU (Figure 1). Duodenoscopy confirmed that the mass originated from the duodenal papilla and projected into the lumen of the duodenum without invading the intestinal wall (Figure 2A). Endoscopic biopsy of the tumor showed a proliferation of polygonal cells, which were a dense arrangement and accompanied by visible mitosis and pleomorphic giant cells. Tumor cells were positive for CD68, Ki67 (50%), and Vimentin (Figures 2B–E), while negative for CD117, CD20, CD3, CD34, chromogranin A, CK7, Desmin, DOG1, HMB-45, melon-A, and Synaptophysin (data not provided). Then, the diagnosis considered a high-grade sarcoma, and a complete resection of the tumor was recommended for further examination.

**Figure 3:**
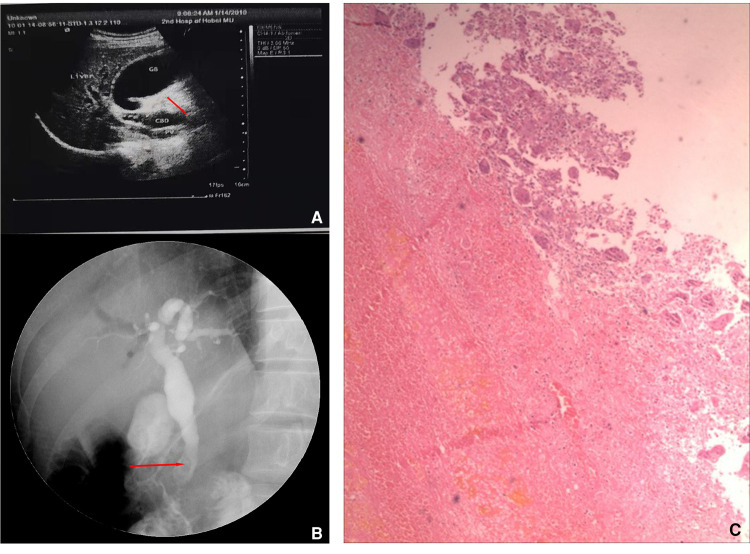
Ultrasound, endoscopy, and biopsy results 8 years ago. Preoperative ultrasound (**A**) and endoscopic retrograde cholangiopancreatography (ERCP) images showed a mass (red arrow) in the distal bile duct (**B**), and the histological manifestation of biopsy specimen (**C**).

What is noteworthy is that the patient was admitted to our hospital 8 years ago with obstructive jaundice (bilirubin = 286 μmol/L, especially direct). Ultrasound and endoscopic retrograde cholangiopancreatography (ERCP) were performed at that time, both of which indicated mass in the distal common bile duct (Figures 3A, B). A biopsy specimen obtained from the mass revealed blood clots and multinucleated giant cell reaction (Figure 3C). Subsequently, the patient underwent cholecystectomy, choledochectomy, and Roux-en-Y hepaticojejunostomy. No mass was found in the bile duct during the operation, but stenosis was observed in the pancreatic segment of the common bile duct with a thickness of 0.3 cm. The distal bile duct was resected approximately 0.6 cm below the stenosis. Postoperative pathology showed chronic inflammation of the bile duct with mild hyperplasia of glandular epithelium and adenomyosis. The patient was asymptomatic for 8 years.

**Figure 4:**
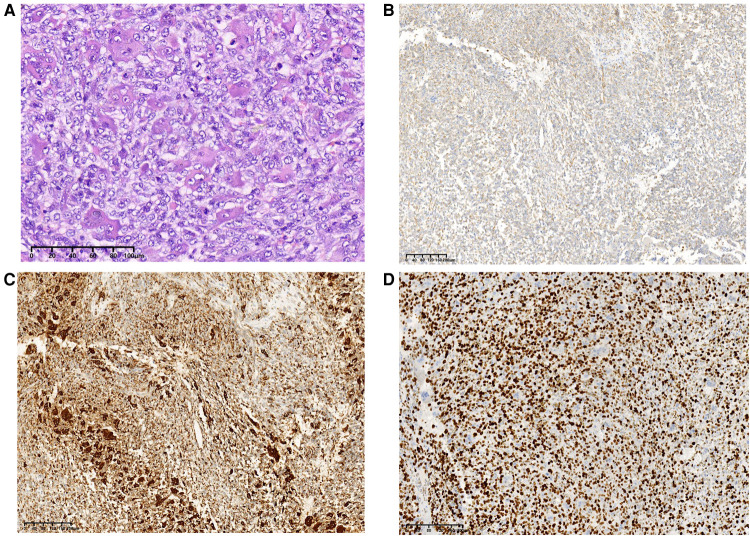
Final pathological results after surgery. Histopathological examination of the tumor (H&E staining), original magnification, ×200 (**A**). These cells were positive for Vimentin, ×100 (**B**), CD68, ×100 (**C**), and Ki-67 was about 80% upon immunostaining, ×100 (**D**).

Based on medical history and the biopsy results during the second admission, the final surgical method was determined as duodenal papilla and tumor resection, rather than pancreaticoduodenectomy. The histopathological findings revealed an UPS. Microscopically, multiple multinucleated osteoclast-like giant cells were evenly distributed on the mononucleated stromal cells. Giant cells contained numerous nuclei with eosinophilic cytoplasm. The pleomorphic stromal cells were spindle-shaped and atypical round, which were similar to the high-grade sarcoma. Nucleoli could be seen in several stromal cells. A small number of stromal cells showed a high degree of mitosis, which is a sign of malignancy. Immunohistochemical stains for CD68 and Vimentin were positive in the neoplastic cells, the proliferative marker Ki67 showing approximately 80% nuclear staining (Figure 4), and CD117, CD34, HMB45, CD45, CD56, S-100, smooth muscle actin (SMA), or desmin were negative (data not provided). The patient declined further chemotherapy and was discharged.

**Figure 5:**
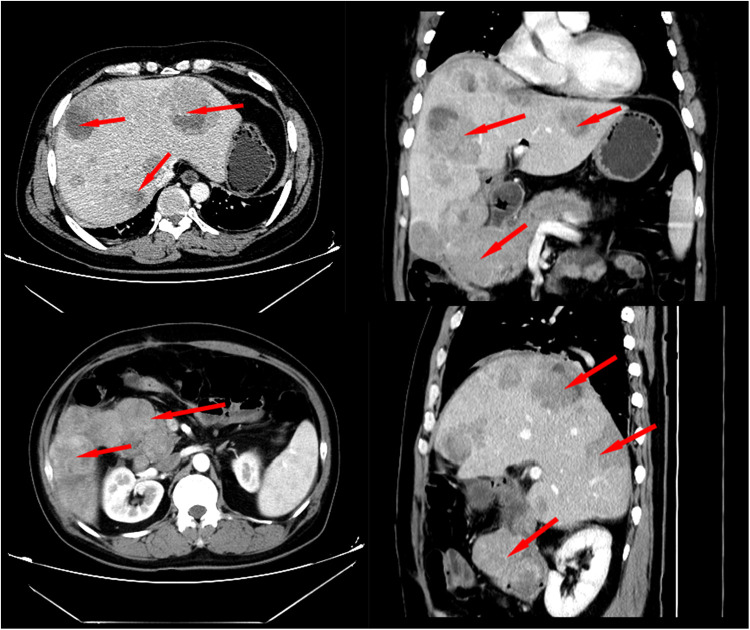
Contrast-enhanced CT scan 2 months after surgery revealed local recurrence and intrahepatic metastasis.

**Figure 6:**
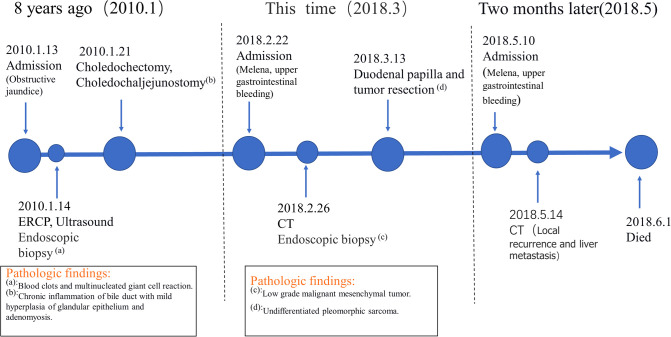
A timeline with relevant data from the episode of care.

Two months later, the patient was admitted to our hospital again with complaints of melena. CT scan showed a recurrent duodenal mass and multiple metastatic foci in the liver (Figure 5), and the patient died of metastasis 2 weeks later. The episode of care is organized as a timeline in Figure 6.

## Discussion

UPS, or previously known malignant fibrous histiocytoma (MFH), is considered the most common type of soft tissue sarcoma. It often occurs in the limbs, trunk, and retroperitoneal tissues (3, 22), but it has been rarely observed in the digestive organ (15). The nomenclature and categorization of this neoplasm have changed several times over the years. In 2002, the World Health Organization (WHO) believed that MFH should be synonymous with UPS and divided into three subtypes, polymorphic MFH/UPS, giant cell MFH/with giant cell UPS, and inflammatory MFH/ with prominent inflammation UPS (23). In 2013, the WHO removed MFH and replaced it with UPS, which was classified into the newly established undifferentiated soft tissue sarcoma (USTS), a group of polymorphic heterogenous stromal tumors with no clear differentiation direction (24, 25).

UPS located at the same site often have similar clinical features. UPS in the head of the pancreas is characterized by epigastric pain, nausea, and vomiting. Some patients may present with weight loss and abdominal mass. Jaundice is also possible, depending on whether the tumor is compressing or invading the bile ducts. Primary UPS of the duodenum is often invasive and can penetrate the intestinal wall, leading to perforation or ulceration bleeding (20). Therefore, gastrointestinal bleeding and melena are the main symptoms of primary UPS in the duodenum. The symptoms caused by UPS arising in the Vater papilla region are similar to those caused by other tumors in this location and include melena, recurrent acute pancreatitis, jaundice, and abdominal pain. Similar to previous cases, melena was the main symptom accompanied by upper abdominal discomfort in this case. The difference is that this patient had undergone a Roux-en-Y hepaticojejunostomy, so jaundice was not present. The histopathology confirmed the diagnosis of UPS.

Patients with UPS in the duodenum often die months or years after diagnosis (18, 20). It is not clear whether the tumor has undergone a long growth process or grown rapidly from the beginning. The endoscopic biopsy result of 8 years ago showed blood clots and multinucleated giant cell reaction in this case, which could not be confirmed as UPS, giant cell tumor, or foreign body giant cell reflection now. A similar case has been reported in the central nervous system where a mass was found in the same area on a plain CT scan 5 years before the diagnosis of primary UPS in the brain (26). In addition, traumatic factors such as surgery are also one of the possible causes of UPS (27). The patient had undergone cholecystectomy and choledochectomy 8 years ago, and traumatic factors may have contributed to the occurrence of UPS in this rare site.

The diagnosis of UPS is important in its treatment process. UPS is defined as a group of sarcomas in which any attempt to disclose their line of differentiation has failed (28). Their immunophenotype is plastic and they may focally express CD34, SMA, and CD68. Immunohistochemistry can be used as a means to rule out melanoma, sarcomatoid carcinoma, and malignant lymphomas, which have respective tumor markers (29), but its role is still auxiliary. The diagnosis continues to presuppose thorough sampling and evaluation of hematoxylin–eosin-stained sections (29). Another differential diagnosis of this case is giant cell tumor of soft tissue because of the existence of multinucleated giant cells (30, 31). However, considering the patient's high Ki67 index (80%) and rapidly progressing history, our final pathological diagnosis was UPS rather than a giant cell tumor of soft tissue (30, 32).

The surgical procedure, in this case, is different from the previous cases. Treatment with UPS emphasizes early complete resection of the tumor to obtain an adequate margin and reduce tumor recurrence. There is no consensus on what is an adequate margin distance for minimizing the risk of local recurrence. When UPS occurs in the extremities, the resection margin should be at least 1–3 cm away from the gross border (33, 34). However, if UPS is located in the retroperitoneum, complete compartmental resection will produce significantly better results (34, 35). Due to the low risk of lymphatic metastasis, routine lymph node dissection is not recommended during surgery (36). In this case, the tumor protruded into the intestinal lumen without invading the intestinal wall, and the intraoperative frozen section results showed a negative resection margin. Therefore, only the tumor and duodenal papilla were removed in this operation, instead of pancreaticoduodenectomy.

## Conclusion

Primary UPS of the duodenal papilla is a highly malignant sarcoma with a poor prognosis. There are no specific clinical manifestations, which may only be jaundice with upper abdominal discomfort. When the tumor is enlarged and necrotic, upper gastrointestinal bleeding such as melena may occur. This tumor represents a challenge in management due to the lack of data in the literature. Early radical surgery is the key to better efficacy. The prognosis was poor despite the early complete resection. Early endoscopy in elderly patients with upper abdominal discomfort may yield early detection of this rare tumor, which needs to be verified in more cases.

## Data Availability

The original contributions presented in the study are included in the article/Supplementary Material; further inquiries can be directed to the corresponding author.
